# Mammary extracellular matrix directs differentiation of testicular and embryonic stem cells to form functional mammary glands *in vivo*

**DOI:** 10.1038/srep40196

**Published:** 2017-01-10

**Authors:** Robert D. Bruno, Jodie M. Fleming, Andrea L. George, Corinne A. Boulanger, Pepper Schedin, Gilbert H. Smith

**Affiliations:** 1 School of Medical Diagnostic & Translational Sciences, College of Health Sciences, Old Dominion University, Norfolk, VA 23529, USA; 2 Department of Biological and Biomedical Sciences, North Carolina Central University, Durham, NC, 27707, USA; 3 Mammary Stem Cell Biology Section, Basic Research Laboratory, CCR, NCI/NIH, Bethesda MD, 20892, USA; 4 Oregon Health and Science University, 3181 SW Sam Jackson Park Road, Portland, OR 97239, USA

## Abstract

Previously, we demonstrated the ability of the normal mammary microenvironment (niche) to direct non-mammary cells including testicular and embryonic stem cells (ESCs) to adopt a mammary epithelial cell (MEC) fate. These studies relied upon the interaction of transplanted normal MECs with non-mammary cells within the mammary fat-pads of recipient mice that had their endogenous epithelium removed. Here, we tested whether acellular mammary extracellular matrix (mECM) preparations are sufficient to direct differentiation of testicular-derived cells and ESCs to form functional mammary epithelial trees *in vivo*. We found that mECMs isolated from adult mice and rats were sufficient to redirect testicular derived cells to produce normal mammary epithelial trees within epithelial divested mouse mammary fat-pads. Conversely, ECMs isolated from omental fat and lung did not redirect testicular cells to a MEC fate, indicating the necessity of tissue specific components of the mECM. mECM preparations also completely inhibited teratoma formation from ESC inoculations. Further, a phenotypically normal ductal outgrowth resulted from a single inoculation of ESCs and mECM. To the best of our knowledge, this is the first demonstration of a tissue specific ECM driving differentiation of cells to form a functional tissue *in vivo*.

Understanding the microenvironmental factors that control cell function, differentiation, and stem cell renewal are at the crux of developmental and cancer biology. In recent years, our laboratory has explored the capacity of the local mammary microenvironment (niche) to control the cellular fate and differential potential of cells^1^,^2^,^3^,^4^,^5^,^6^,^7^,^8^,^9^. Our previous reports have found that the regenerating mouse mammary gland can re-direct testicular cells^1^, neural stem cells^2^, bone marrow^5^, and breast cancer cells^3^,^4^ to adopt a normal mammary progenitor cell fate. Further, we found that the mammary microenvironment can suppress teratoma formation and direct differentiation of mouse embryonic stem cells (ESCs)^7^ and human teratocarcinoma (Ntera-2) cells^3^ to a mammary epithelial cell fate *in vivo*. In all cases, the redirected non-mammary or cancer-derived cell types continued to proliferate and self-renew, persisting through multiple transplant generations. All of these studies were performed with essentially the same experimental design: dispersed normal mouse mammary epithelial cells were mixed with non-mammary or cancer cells and inoculated into the cleared mammary fat-pad of a recipient mouse. Our interpretation of this is that the non-mammary and cancer-derived cells are incorporated into mammary niches, due to cell-cell interaction, as these locations are formed by the dispersed MECs during growth. Once incorporated, they adopt a normal mammary cell fate and contribute mammary progeny to the outgrowth. These studies underscore the dominance of the mammary locale (niche) in determining cell fate in the mammary gland. However, the nature and composition of the mammary niche(s) remains elusive.

The extracellular matrix (ECM) is a key component of the cellular microenvironment. More than just a scaffold of support proteins, the ECM is tethered with diverse signaling molecules that show tissue-specific patterning^10^,^11^. In the mammary gland, the ECM is dynamic, changing in both composition and structure with pregnancy, lactation, and involution^12^. ECM has a role in stem cell renewal and differentiation both *in vivo* and *in vitro*, and thus is likely an important mediator of the mammary stem cell niche.

Here, we tested the hypothesis that mammary ECM contains factors that are integral components of the mammary niche and thus sufficient to direct differentiation of non-mammary cells in the context of the mammary fat-pad. Specifically, we tested if mammary ECM (mECM) extracts would be sufficient (in the absence of normal MECs) to direct the differentiation of testicular and ESCs to MECs when transplanted into a cleared mammary fat-pad. Testicular cells were sourced from WAP-CRE/Rosa26-loxp-stop-loxp-LacZ (WC/R26LacZ)^13^. These mice have been previously described to mark a particular progenitor population termed parity identified mammary epithelial cells (PI-MECs) following pregnancy and involution^6^,^14^,^15^,^16^. Briefly, activation of the whey acidic protein (WAP) promoter during pregnancy induces CRE expression which in turn removes a premature floxed stop-codon causing irreversible and constitutive β-galactosidase (β-gal) expression from the Rosa26-LacZ locus. PI-MECs are found among a fraction of luminal cells within secondary and tertiary ducts, and function as lobular progenitors in subsequent pregnancies^14^,^15^. The ESCs used here were isolated from Rosa26-LacZ mice, and constitutively express β-gal in all cell types^17^,^18^. These two reporters allowed us to identify the exogenous cell sources in resulting transplants by 5-bromo-4-chloro-3-indolyl-β-D-galactopyranoside (X-gal) staining. mECM was isolated from mammary glands of adult female nulliparous mice, as well as nulliparous and involuting rats. We identified X-gal+ outgrowths in a total of 26/99 mammary glands inoculated with testicular cells and mECM across all conditions. The outgrowths displayed normal mammary gland morphology and milk protein production. Conversely, ECM from omental fat or lung were incapable of directing testicular cells to form mammary outgrowths. We also found that mECM eliminated teratoma formation of ESCs and resulted in an X-gal+ outgrowth in a single case. The resulting outgrowth demonstrated cytokeratin and hormone receptor expression. These results demonstrate that the non-cellular mECM fraction of the mammary gland contains critical factors of the mammary niche that, in conjunction with the mammary fat-pad, are sufficient to direct non-mammary cells to a mammary epithelial cell fate. These findings are, to the best of our knowledge, the first demonstration of the capacity of adult tissue specific ECM to mediate the direct differentiation of stem cells and inhibit teratoma formation *in vivo*.

## Results

### Mouse mammary ECM (mECM) redirects testicular cells to adopt a normal mammary epithelial cell fate

**Figure 1:**
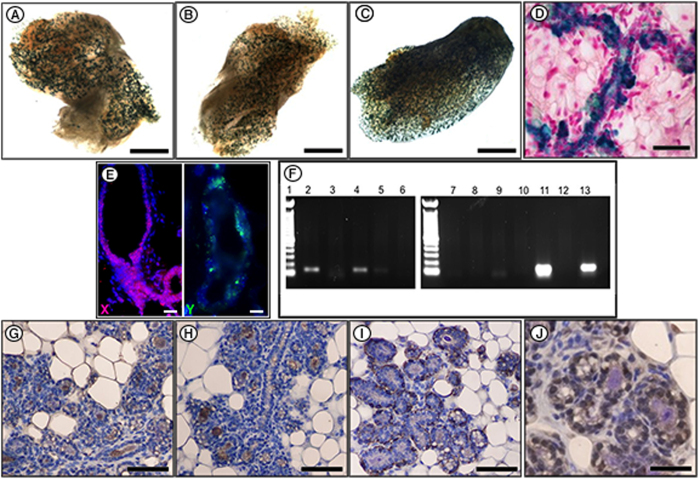
Testicular cells form normal mammary outgrowths *in vivo* when transplanted with mouse mECM. 7.5 × 10^4^ testicular cells derived from WC/R26-LacZ mice were inoculated in suspensions of mECM, omental fat ECM, lung ECM, or vehicle (DMEM) into cleared mammary fat-pads of female nude mice. Following full-term pregnancy and 3 weeks of involution to activate the WC/R26-LacZ reporter in the testicular-derived cells, glands were isolated for analysis. (**A**–**C**) Examples of X-gal+whole mounts from inoculations of testicular cells and mECM. X-gal+outgrowths were not seen in any of the testicular cells + control (omental fat ECM, lung ECM, and DMEM) groups. (**D**) Cross-section of a an X-gal stained gland derived from testicular cells. X-gal stain is blue, nuclei are counterstained with nuclear fast red. (**E**) FISH analysis of testicular derived outgrowths with probes to the X-chromosome (magenta; left panel) and Y chromosome (green, right panel). (**F**) PCR with primers specific for the Y chromosome. Lane 1: Molecular weight marker; Lane 2, 4, and 5: Testicular cells + mECM outgrowth; Lane 3: Testicular cells + mECM inoculated fat pad with no outgrowth; Lane 6: water; Lane 7&8: MEC + mECM outgrowths; Lane 9&11: outgrowth derived from MEC + testicular cells; Lane 10: MEC + testicular cell inoculated fat pad with no outgrowth; Lane 12: MEC cells; Lane 13: Testicular cells. (**G–J**) IHC staining for alpha-lactalbumin (**G**), caseins (**H**), smooth muscle actin (**I**), and ERα (**J**) in outgrowth of testicular cells and mECM in 14 day pregnant host (nuclei counterstained with haematoxylin). Scale bars: **A**–**C** = 2 mm; **D** = 200 μM; **E** = 100 μM; **G**-**I** = 100 μM; **J** = 200 μM.

Cells isolated from the testes of WC/R26Lacz mice were the first to be shown to respond to signals within the regenerating mouse mammary gland to adopt a normal mammary epithelial progenitor cell fate^1^. To advance these findings, we tested whether physical contact with mammary epithelial cells was required, or whether the soluble and structural constituents of the mECM were sufficient to redirect testicular cells to a mammary epithelial cell fate. 7.5 × 10^4^ mouse testicular cells were injected with or without soluble mECM isolated from adult nulliparous female BALB/c mice into the cleared mammary fat pads of recipient female nude mice. Following pregnancy and involution, whole mount and cross-section imaging of mammary glands revealed normal X-gal+ mammary epithelial outgrowths in 16/62 inoculations with mECM (Table 1; Fig. 1A–D). Importantly, the pattern of X-gal staining was consistent with the expected distribution of PI-MECs in intact WC/R26-LacZ, suggesting normal activation of the Wap promoter. Consistent with previous results^1^, inoculations of testicular cells in vehicle (DMEM) did not generate mammary outgrowths (0/15; Table 1). Further, no Xgal+ outgrowths were observed when testicular cells were inoculated with matrigel (not shown) or with ECM isolated from omental fat (0/14) or lungs (0/19) of adult mice (Table 1). The difference in rate of outgrowth formation between inoculations of testicular cells with mECM or with omental fat ECM, lung ECM or vehicle (DMEM) was statistically significant (p = 0.0327; p = 0.0173, and p = 0.0326, respectively). Therefore, the factors critical for the differentiation of the testicular cells to MECs are specific to mECM. We identified the presence of the Y chromosome by fluorescent *in situ* hybridization (FISH) and PCR in the outgrowths, confirming they were derived from the testicular cells and not ingrowth of endogenous epithelium (Fig. 1E and F). Furthermore, inoculations of the cleared fat pads with mECM alone did not induce outgrowths (0/10; Table 1) or notable changes to the adipose/stroma.

It is important to note that positive X-gal staining under these experimental conditions is only seen in the mammary gland upon exogenous expression of β-gal^13^,^19^. Exogenous β-gal expression could only come from the transplanted testicular cells. Further, to express β-gal, the testicular cells must have activated the mammary specific WAP promoter during pregnancy and subsequently survived involution. This is consistent with the interpretation that the testicular derived cells had differentiated into fully functional mammary epithelial cells including PI-MECs.

To determine if the testicular-derived epithelial trees were capable of normal MEC function, fragments taken from first generation outgrowths were transplanted into cleared mammary fat pads of new hosts and the mice were mated to induce lactogenic differentiation. At 14 days of pregnancy, glands were removed and cross sectioned. Staining with antibodies specific for the milk proteins alpha-lactalbumin and caseins revealed normal milk protein production and luminal secretion, consistent with normal mammary epithelial cell function (Fig. 1G and H). The glands also expressed the basal myoepithelial cell marker smooth muscle actin alpha (SMA; Fig. 1I) and the hormone receptor ERalpha (ERα; Fig. 1J) in the correct orientations. Combined, these results demonstrate that the testicular derived cells had made a fully functional mammary epithelial tree upon transplantation with mECM.

### mECM from both nulliparous and involuting rats redirect testicular cells to adopt a normal mammary epithelial cell fate

**Figure 2:**
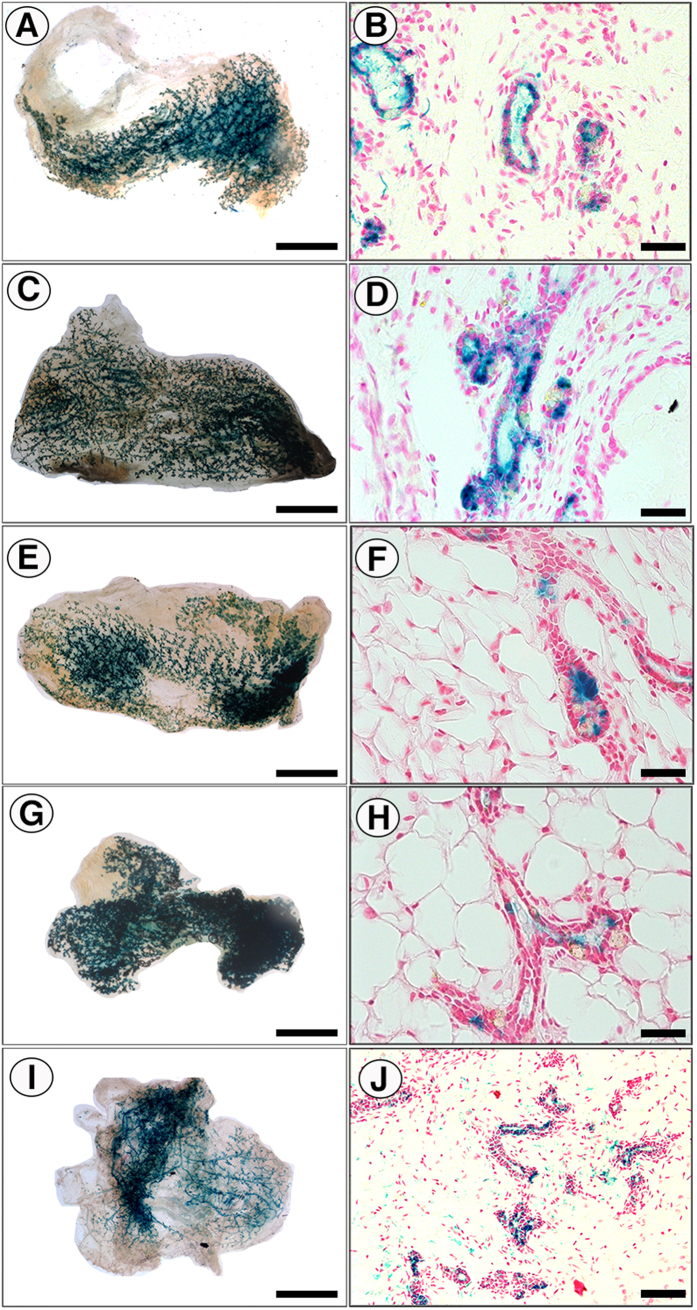
Testicular cells are directed to MECs by both nulliparous and involuting rat mECM *in vivo*. 5.0 × 10^4^ testicular cells from WC/R26-LacZ mice were inoculated in suspensions of nulliparous rat mECM, involuting rat mECM, or vehicle (DMEM) into cleared mammary fat-pads of female nude mice. Following full-term pregnancy and 3 weeks of involution to activate the WC/R26-LacZ reporter in the testicular-derived cells, glands were isolated for analysis. (**A**–**D**) X-gal stained whole mounts (**A** and **C**) and cross-sections (**B** and **D**) of mammary outgrowths derived from testicular cells and nulliparous rat mECM. (**E**–**H**) X-gal stained whole mounts (**E** and **G**) and cross-sections (**F** and **H**) of mammary outgrowths derived from testicular cells and involuting rat mECM. (**I** and **J**) X-gal stained whole mount (**I**) and cross-section (**J**) of second-generation outgrowth derived from transplantation of tissue fragments of primary outgrowths of testicular cells and nulliparous mECM. X-gal+stain is blue. All cross-sections are counterstained with nuclear fast-red. Scale Bars: **A**,**C**,**E**,**G** and **I** = 2 mm; **B**,**D**,**F**, and **H** = 200 μM; **J** = 400 μM.

Following these initial observations, we next turned to mECM isolated from Sprague-Dawley rat mammary tissues. Rat mammary epithelial cells grow normally in mouse fat pads and mouse cells respond to rat mECM^12^,^20^. Rat tissue is advantageous because of the greater concentrations of mECM that can be produced from the larger rat glands. 5 × 10^4^ WC/R26-LacZ mouse testicular cells were injected with or without soluble mECM from nulliparous or involuting female rats into the cleared mammary fat-pad of nude recipient female nude mice. As previously reported^1^, 5 × 10^4^ testicular cells never formed glands when inoculated alone (0/20; Table 1). Both nulliparous ECM and involuting ECM preparations were used because previous studies have identified differences in their content and stimulatory effects on breast cancer cells^21^,^22^. Following pregnancy and involution, whole mount and cross-section imaging of mammary glands revealed normal X-gal+ mammary epithelial outgrowths in 4/18 inoculations with nulliparous mECM (p = 0.0415 vs testicular cells alone) and 6/19 inoculations with involuting mECM (p = 0.0083 vs. testicular cells alone; Fig. 2; Table 1). There was no statistical difference in the effect of involuting mECM vs nulliparous mECM (p = 0.7140).

An important feature of the mouse mammary gland is the capacity of any portion of the epithelial tree to regenerate a functional mammary tree upon transplantation^23^. To determine if the testicular/mECM derived outgrowths retained this capacity, we transplanted fragments from first-generation outgrowths resulting from inoculation of testicular cells and nulliparous mECM into cleared mammary fat-pads and stained the resulting glands with X-gal. 5/8 transplants resulted in mammary outgrowths that contained X-gal+ cells, with the remaining 3 not forming any outgrowth (Fig. 2I and J; Table 1). This is consistent with previous results that demonstrated that non-mammary cells redirected to a MEC fate by the mammary microenvironment could contribute to second-generation outgrowths^2^,^5^,^7^,^16^. Presence of the X-gal+ cells in second generation outgrowths demonstrates that the β-gal+ testicular derived cells were not terminally differentiated, but capable of self-renewing and undergoing extensive proliferation to regenerate the gland.

### Mammary ECM extracts inhibit teratoma formation and direct differentiation of mouse ES cells to mammary epithelial cells in cleared mammary fat-pads

**Figure 3:**
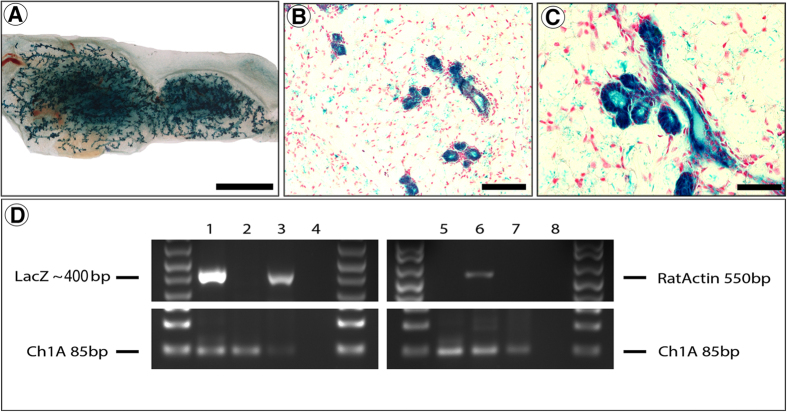
Normal mammary outgrowth derived from ESCs and involuting rat mECM. In 1/5 inoculations of 1 × 10^3^ R26-LacZ ESCs and involuting rat mECM a morphologically normal x-gal+mammary outgrowth was observed. (**A**–**C**) X-gal+whole mount (**A**) and cross-sections (**B** and **C**) of ESC-derived mammary outgrowth. (**D**) PCR analysis using primers specific for LacZ gene, rat actin, and mouse chromosome 1 (positive control) in the outgrowth shown in **A**–**C**. Lane 1 = R26-LacZ mouse tail DNA; Lane 2: WT Balb/c mouse tail DNA; Lane 3: ESC + ECM outgrowth DNA; Lane 4: Water; Lane 5: WT Balb/c mouse tail DNA; Lane 6: Rat LA-7 cell DNA; Lane 7: ESC + mECM outgrowth DNA; Lane 8: Water. Results confirm outgrowth is comprised of R26-LacZ mouse ESC-derived cells. Scale Bars: **A** = 1.5 mm; **B** = 100 μm; **C** = 200 μm.

We next sought to determine if pluripotent ESCs could similarly be directed to a MEC fate by inoculation with mECM into cleared mammary fat-pads. We injected 1 × 10^3^ or 1 × 10^4^ Rosa26-LacZ ESCs (which constitutively express β-gal) with or without involuting ECM. As previously reported^7^, Rosa26-LacZ ESCs formed teratomas in all cases when inoculated in vehicle (DMEM) alone (Table 2). However, in the presence of mECM, none of the ESC inoculations formed teratomas (Table 2). This reduction in teratoma formation by mECM is statistically significant (p = 0.0079) for both cell injection numbers (1 × 10^3^ and 1 × 10^4^). Further, 1/5 inoculations of 1 × 10^3^ Rosa26-LacZ ESCs formed a mammary epithelial outgrowth that stained positive for X-gal (Fig. 3A). Cross sections demonstrated that the Xgal+ cells were present throughout the ductal tree in nearly all cells (Fig. 3B and C), as expected for the constitutive Rosa26-LacZ reporter.

To further verify the mammary epithelial tree consisted of the transplanted Rosa26-LacZ ESCs and did not result from ingrowth of endogenous cells, we performed PCR on DNA isolated from the outgrowth to detect the Rosa26-LacZ locus (Fig. 3D). Primers specific for the Rosa26-LacZ locus amplified DNA in the X-gal positive outgrowth shown in Fig. 3A but not in control mouse glands. Further, primer sets specific for rat DNA confirmed no contamination of rat MECs within the mECM.

**Figure 4:**
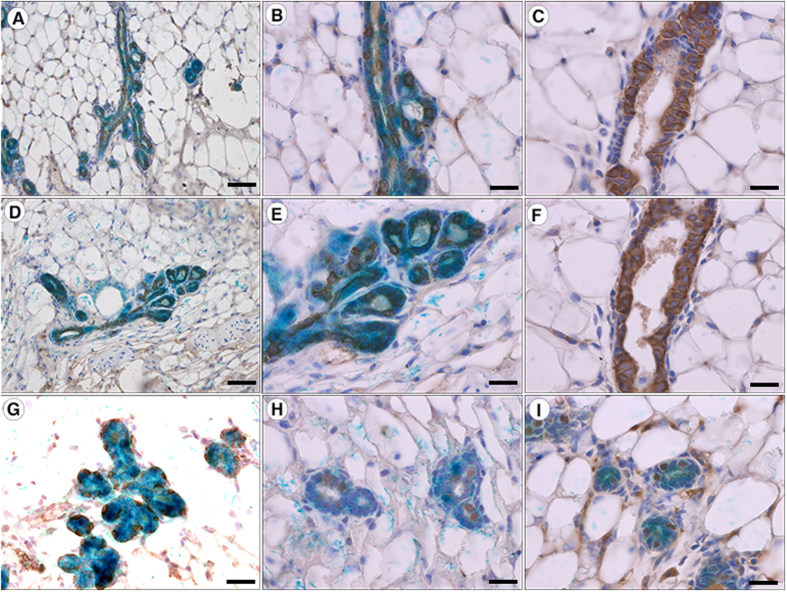
Expression of MEC proteins in ESC-derived mammary outgrowth. (**A**–**C**) Cross-sections of ESC-derived outgrowth (**A** and **B**) and control gland (**C**) were stained with a an an anti-pan-cytokeratin antibody. (**D**–**F**) Cross-sections of ESC-derived outgrowth (**D** and **E**) and control gland (**F**) were stained with an anti-cytokeratin 8 antibody. (**G–I**) ESC derived mammary outgrowth stained with an anti-SMA (**G**), anti-ERα (**H**), and anti-progesterone receptor (**I**). X-gal stain is seen in blue. Sections are counterstained with haematoxylin. Scale bars: **A**, **D**, and **G** = 200 μM; **B**,**C**,**E**,**F**,**H** and **I** = 100 μM.

Finally, we immunostained the resulting mammary outgrowth to determine if the ESCs had differentiated into all the major epithelial cell types of the mammary gland. The outgrowth stained positive with a pan-cytokeratin antibody (Fig. 4A and B) although the staining lacked uniformity as usually seen in normal mammary tissues (Fig. 4C). Similarly, staining with an antibody specific for the luminal marker cytokeratin 8 (Fig. 4D and E) demonstrated the presence of CK8 cells with a lack of uniformity compared to control glands (Fig. 4F). Conversely, normal staining confined to the basal layer was seen with the basal marker SMA (Fig. 4G). Further, ERα and progesterone receptor (PR) positive and negative cells were seen throughout the luminal layer of the ESC-derived gland (Fig. 4H and I). The reason for the punctate staining pattern of the cytokeratins is unknown. It could either be a result of incomplete differentiation of some of the ESC-derived cells or an artifact of the sample preparation. The normal staining seen in a control sample within the same block argues against the latter interpretation, however. Regardless, these results demonstrate that the Rosa26-LacZ derived ESCs were capable of differentiating into all of the major mammary epithelial cell types when injected along with mammary ECM into the cleared mammary fat-pad.

## Discussion

While a great deal of focus has been paid to the identification and characterization of somatic cells with stem cell characteristics, we continue to lack a basic understanding of how heterogenous lineages of cells are generated and maintained *in vivo*. Our recent work has demonstrated that in the mammary gland, the components of the microenvironment (i.e. niche) are the ultimate determinant of cell function, regardless of the original nature of the stem/progenitor cells within the system. Here we extend these observations by demonstrating that the acellular mECM fraction of the gland contains key components of the niche that are sufficient to direct differentiation of non-mammary cells to a MEC fate in the context of the mammary fat-pad *in vivo*. In the case of the testicular derived cells, they had all of the attributes associated with normal MECs, including the capacity to make milk proteins (Fig. 1G and H) and persist through second-generation outgrowths (Fig. 2I and J). This is important because it demonstrates the signaling effect of the mECM is not to terminally differentiate the cells, but rather assist in the organization of the cells into fully functional mammary niches, capable of supporting all aspects of gland development and function. One important distinction between the present study and our previous publications is that the non-mammary cells were able to differentiate and form the entire functional mammary gland without assistance from bona fide MECs. This in itself is remarkable because it indicates that all the signals necessary to form a functional mammary epithelium in a competent mammary fat-pad are present in the “ECM’ preparations from intact mammary glands.

The other major finding of this work is that mECM from involuting mammary glands significantly reduced teratoma formation by the ESCs (Table 2). In fact, under our conditions, teratoma formation was completely eliminated. This is in contrast to previous publications demonstrating pro-tumorigenic affects of involuting mECM on breast cancer cells^22^. This difference may be due to differential effects of mECM on pluripotent ESCs versus breast cancer cells, with the former potentially responding by differentiation and/or apoptosis to the presence of the mECM. It should be noted, no evidence of differentiation of ESCs—that is presence of X-gal+ cells—was seen in the fat-pads of the nine inoculations that did not form teratomas or mammary outgrowths. This suggests that the ESCs had not survived the 12 weeks *in vivo*. However, the presence of a small amount of ESCs cannot be ruled out.

The mechanism(s) by which mECM mediates the suppression of teratoma formation and the differentiation of testicular and ESCs to mammary epithelial cells is currently unknown. Two possibilities exist: 1. The mECM directly induces the differentiation of the non-mammary cells to MECs, which in turn communicate with the stromal fat-pad environment to develop into a mammary epithelial tree; 2. The mECM activates signaling within the stromal fat-pad environment to produce signals that direct the differentiation of the non-mammary cells to differentiate to MECs. The latter is more likely, but future studies should determine the direct effects of mECM on both the non-mammary cells (outside of the mammary fat-pad) and the stromal cells within the mammary fat-pad. Further, specific components of the mECM preparations should be evaluated to determine the critical elements of the mECM. Because the low frequency of gland formation with both testicular and ESC inoculations with the soluble mECM preparations used, future studies should also evaluate the effect of different mECM preparations (e.g. pepsin digestion) that result in differential protein compositions and structures. Future studies might also evaluate the effect of different mECM preparations after pretreatment with proteases or nucleases to help evaluate the nature of the active substances.

Given the wealth of literature showing the unique proteinaceous composition of the mammary ECM, which also alters during distinct developmental stages^12^,^20^,^24^,^25^,^26^,^27^,^28^, pinpointing the exact molecules and the orchestration of signaling between the stroma and parenchyma to direct cell behavior is a challenge. ECM isolated from nulliparous rats has been shown to promote the formation of epithelial ducts with bifurcation, while matrix isolated from mid-involuting mammary glands induced cell death. ECM isolated from late-stage involuting glands restored glandular development, and ECM isolated from parous animals restricted glandular morphogenesis^12^. Our studies support this work by demonstrating both nulliparous and late-stage involuting ECM promote growth and reprogramming of testicular and embryonic cells to mammary epithelial cells. Collectively, these studies highlight that hormones, cytokines, and growth factors all influence ECM composition including collagen organization and stiffness, to affect mammary cell behaviors such as organization and function of integrins and the expression of cell-cell junction proteins and matrix metalloproteinases^25^. Critical to data presented within, recent studies suggest that it is not individual molecules, but the relative abundance and organization of the ECM molecules that dictate tissue function and regulation of cell fate. For example, Goddard *et al*. demonstrated that the mammary gland and liver have the same ECM components, but distinct abundance and relative composition of individual protein components^28^. Ultimately, both mechanical and biochemical aspects of the soluble and insoluble ECM must be integrated to understand the collective function. Therefore, the mechanism(s) governing ECM mediated control over cell fate determination are likely to be complex, and not governed by single “magic bullet” components.

Regardless of the mechanism(s), these findings have potentially major implications for our understanding of stem cell biology, cancer biology, and regenerative medicine. The mammary niche is composed of both mammary epithelial cells and the stromal microenvironment of the mammary fat-pad^9^,^23^. Together, this niche is sufficient to direct fate determination of non-mammary and cancer cells to make a functional mammary epithelial tree. The results outlined here demonstrate that the mECM is a key mediator of the niche. The experimental protocol can be used to test individual components of the mammary niche and shed light on mediators of normal development and cancer. Finally, the ability to of the mECM to reduce teratoma formation and potentially direct differentiation of pluripotent cells could benefit regenerative medicine applications, particularly to mitigate fears of teratoma formation in clinical applications.

These studies demonstrate that mammary epithelial cells are dispensable for the redirection of non-mammary cells to adopt a MEC fate *in vivo*. Further, the signaling required to redirect testicular cells to a MEC fate are specific to mECM, as ECM isolated from omental fat or lung failed to mediate lineage conversion. To our collective knowledge, this is the first demonstration that cell fate may be altered *in vivo* by tissue specific ECM. The significance of this observation is that it opens the possibility of altering cell fate decisions *in vivo* without the use of cells or chemicals and has an important potential role in the control and prophylaxis of mammalian cancers *in situ*. The results allow for mechanistic dissection of key elements of the mammary microenvironment and have important implications for our understanding of developmental biology, cancer, and regenerative medicine.

## Methods

### Animal Models

All methods were performed in accordance with the NIH Guide for the Care and Use of Laboratory Animals. The National Cancer Institute (NCI) Animal Care and Use Committee approved all experimental procedures. Mature BALB/c and Sprague Dawley rats were used as donor mice to obtain ECM extracts. Female, 3-week-old athymic Nu/Nu mice were used as hosts for transplant studies (NCI Animal production area; Frederick, MD).

### Cell Isolation

Testicular cells were isolated from adult male WAP-CRE/Rosa26-loxp-stop-loxp-LacZ (WC/R26LacZ) mice as previously described^1^,^8^. Isolated testicular cells were inoculated immediately following isolation. Cells were counted by hemacytometer and diluted in appropriate volumes of either soluble mammary ECM or plain Dulbecco’s Modified Eagle Medium (DMEM).

ESCs isolated from the Rosa26-LacZ mouse were a kind gift from Philipe Soriano^17^,^18^. Cells were cultured as previously described 7. For all experiments, frozen vials of ESCs were rapidly thawed immediately before inoculation *in vivo*.

### ECM Isolation

Acellular extracts were performed as described previously^12^. Briefly, mice/rats were euthanized and the desired tissues were immediately removed, pulverized and extracted with a high-salt/N-ethylmaleimide solution [3.4 M NaCl, 50 mM Tris-HCl (pH 7.4), 4 mM EDTA-Na2, and 2 mM N-ethylmaleimide] containing a protease inhibitor cocktail at 4 °C. Homogenates were pelleted at RCF_max_ 110,000× g for 30 min at 4 °C, and then washed three times in the high salt buffer. The ECM-enriched pellets were resuspended in a mid-level salt/urea solution [2.25 M urea, 0.2 M NaCl, 50 mM Tris-HCl (pH 7.4), 4 mM EDTA-Na2, and 2 mM N-ethylmaleimide] with protease inhibitors and extracted overnight at 4 °C. Samples were pelleted at RCF_max_ 17,000× g, and the ECM-enriched supernatants were extensively dialyzed (Mr 12,000–14,000 molecular weight cutoff; Spectrum) against a low-salt buffer [0.15 M NaCl, 50 mM Tris-HCl (pH 7.4), and 4 mM EDTA-Na2], followed by dialysis against serum-free DMEM:F-12 media supplemented with 1 μg/ml gentamicin at 4 °C. Protein content was measured via Bradford assay. Samples were standardized to equal protein concentrations (mouse ECM = 100 μg/ml; Rat mECM = 300 μg/ml).

### Mammary fat-pad clearing and cell inoculation

Mammary fat pad clearing was performed on female nude mice between 3 and 4 weeks of age as previously described^1^,^7^,^29^,^30^. Briefly, mice were anesthetized and endogenous epithelium was removed from the #4 and #9 inguinal fat pads by surgically excision of the proximal portion (from the nipple to the lymph node) of the gland. 5 × 10^4^ or 7.5 × 10^4^ freshly isolated testicular cells were then inoculated in 10 μl of ECM or plain DMEM (control) into the cleared mammary fat-pad using a Hamilton syringe. After staples were removed 2 weeks post-operation, the recipient mice were mated to activate the WC/R26-LacZ reporter. Following parturition, the pups were euthanized and the mice were allowed to involute for 3 weeks prior to collection of the glands. For ESC transplants, 1 × 10^3^ or 1 × 10^4^ R26-LacZ ESCs were inoculated into cleared mammary fat-pads as described above for testicular cells. Glands were removed after 12 weeks (without mating). All glands were then excised and fixed in 4.0% paraformaldehyde for 2 hrs prior to X-gal staining. Outgrowth and tumor formation rates were compared statistically by Fisher’s Exact Test.

### X-Gal Staining and Immunohistochemistry

X-gal staining and whole mounting of mammary glands was carried out as previously described^1^,^7^,^13^. #3 and #8 thoracic glands from the recipient nude mice were used as negative staining controls for all X-gal staining procedures. X-gal-stained whole mounts were embedded in paraffin, sectioned at 6.0 μm, and counterstained with nuclear fast red (Sigma-Aldrich, St Louis, MO, USA). Primary antibodies used were rabbit-anti-Cytokeratin 8 (ab53280 1:100; Abcam, Cambridge, MA, USA), rabbit-anti-keratin wide spectrum (Z0622 1:500; Dako, Carpinteria, CA, USA), rabbit-anti-ER alpha (sc-542 1:75; Santa Cruz Biotechnology, USA), rabbit-anti-progesterone receptor (A0098 1:150; Dako), and mouse-anti-SMA 1A4 (1:100; Life Technologies, Carlsbad, CA, USA), anti-alpha-lactalbumin^31^ and anti-caseins^32^. IHC staining procedure was carried out using the RTU Vectastain Universal ABC Kit and DAB peroxidase substrate kits (Vector Laboratories, Burlingame, CA, USA) per the manufacturers protocol. All sections were counter-stained with nuclear fast red or haematoxylin.

### FISH Analysis

Dual-color FISH was performed on serial sections. Slides were de-paraffinized by three treatments in xylene and then dehydrated in 100% ethanol. Slides were prepared for pepsin treatment following manufacturer’s instructions from DAKO (Histology Kit, K5599). DAKO pepsin was added at approximately 200 μl per slide. Slides were heated to 37 °C for 10 min on Hybridizer (Abbott Molecular, Thermobrite) with lid open using fixed temperature program. The slides were then washed in 2 × SSC, and dehydrated in an ethanol series and allowed to dry. The chromosome paints were obtained as previously described by chromosome flow sorting^33^, followed by degenerate oligonucleotide primed PCR amplification^34^. The flow sorted probes were labeled with biotin-16-dUTP and digoxigenin-dUTP. *In situ* hybridizations of the probes were performed using 5 μl concentrations of biotin labeled probe and DIG labeled probe. The mixture was precipitated and dissolved in 14 μl of hybridization buffer (formamide 50%, dextran sulfate 10%, 2× SSC). The probe was denatured at 80 °C for 10 min and reannealed at 37 °C for 90 min before hybridization. The previously prepared slide was denatured in 70% formamide/2× SSC, at 65 °C for 80 sec, and quenched in an ice-cold 70% ethanol followed by dehydration in a room temperature 70%, 90%, and 100% ethanol series. Hybridization was carried out in a humidity chamber at 37 °C overnight. Slides were washed and counterstained with diamidino-2-phenylindole (DAPI) (0.8 ng/μl) for 10 min and the slides were mounted with antifade. Analyses were performed under an Axioplan 2 (Zeiss) fluorescence microscope coupled with a CCD camera (Photometrics), and images were captured with FISHview 4.5 software (Applied Spectral Imaging Inc., Vista, CA).

### DNA Isolation and PCR

DNA was isolated from wild type mouse tail tissue, LacZ+ mouse tail tissue, LA-7 rat cells, and mammary tissues using Qiagen DNeasy Blood and Tissue kit (cat # 69506 Qiagen; Valencia, CA, USA). PCR detection was performed using the following primers: SRY primers: 5′-GCTGGGATGCAGGTGGAAAA and 5′-CCCTCCGATGAGGCTGATATT. LacZ primers: 5′-GGATACTGACGAAACGCCTGCC and 5′-GATCCGCGCTGGCTACCGGC; rat actin: 5′-GGCTTTAGGAGCTTGACAATACTG and 5′-GCATTGGTCACCTTTAGATGGA; Control primers to chromosome 1 were previously described^35^.

The amplified products were visualized on a 2% agarose gel containing 500 ng/mL ethidium bromide and illuminated under ultraviolet light. Water served as a negative loading control.

## Additional Information

**How to cite this article**: Bruno, R. D. *et al*. Mammary extracellular matrix directs differentiation of testicular and embryonic stem cells to form functional mammary glands *in vivo. Sci. Rep.* **7**, 40196; doi: 10.1038/srep40196 (2017).

**Publisher's note:** Springer Nature remains neutral with regard to jurisdictional claims in published maps and institutional affiliations.

## Figures and Tables

**Table 1:** Transplantation results for WC/R26-LacZ testicular cells with mECM.

Cell Source	Treatment	Xgal+ mammary outgrowth/total inoculations	Xgal+ second generation outgrowths inoculations
7.5 × 10^4^ Testicular Cells	Nulliparous Mouse mECM	16/62	ND
7.5 × 10^4^ Testicular Cells	Omental Fat ECM	0/14	N/A
7.5 × 10^4^ Testicular Cells	Lung ECM	0/19	N/A
7.5 × 10^4^ Testicular Cells	DMEM	0/15	N/A
None	Nulliparous Mouse mECM	0/10	N/A
5 × 10^4^ Testicular Cells	Nulliparous Rat mECM	4/18	5/8
5 × 10^4^ Testicular Cells	Involuting Rat mECM	6/19	ND
5 × 10^4^ Testicular Cells	DMEM	0/20	N/A

Cell/ECM mixtures were inoculated into the epithelium divested (cleared) mammary fat-pads of 3–4 week old female nude mice. Mice were impregnated and allowed to involute for 3 weeks to activate the WC/R26-LacZ reporter. Second generation outgrowths were generated by transplanting fragments from Xgal+ primary outgrowths into cleared mammary fat-pads of 3–4 week female nude old mice and allowed to grow for 8 weeks. ND = not determined; N/A = not applicable.

**Table 2:** Transplantation results for R26-LacZ ESCs with mECM.

Cell Source	Treatment	Xgal+ mammary outgrowth/total inoculations	Teratoma/total inoculations
1 × 10^3^ ESCs	Involuting Rat mECM	1/5	0/5
1 × 10^3^ ESCs	DMEM	0/4	4/4
1 × 10^4^ ESCs	Involuting Rat mECM	0/5	0/5
1 × 10^4^ ESCs	DMEM	0/4	4/4

Cell/ECM mixtures were inoculated into the epithelium divested (cleared) mammary fat-pads of 3–4 week old female nude mice. Glands were allowed to grow for 12 weeks. Differences in teratoma formation rates between mECM and vehicle (DMEM) treatments were significant (p = 0.0079) for both cell injection numbers (1 × 10^3^ and 1 × 10^4^).
